# Parenting Interventions During the COVID-19 Pandemic: A Systematic Review of the Rationales, Process, Feasibility, Acceptability, and Impacts of Adaptation

**DOI:** 10.1177/15248380241266183

**Published:** 2024-07-31

**Authors:** Zuyi Fang, Mackenzie Martin, Lauren Copeland, Rhiannon Evans, Yulia Shenderovich

**Affiliations:** 1 Peking University, Beijing, China; 2 McMaster University, Hamilton, ON, Canada; 3 Cardiff Metropolitan University, UK; 4 Cardiff University, UK

**Keywords:** parenting intervention, child maltreatment, adaptation, digital delivery

## Abstract

Evidence shows that parenting interventions are an effective method of reducing caregiver-perpetrated child maltreatment. The recent COVID-19 pandemic has changed the provision of parenting interventions worldwide, with many interventions adapting to continue providing services during the crisis. This global systematic review examined how parenting interventions targeting child maltreatment and its risk and protective factors were adapted during the COVID-19 pandemic. We searched for studies published between 2020 and 2022 and identified 31 eligible studies. The data on the rationale, process, feasibility, acceptability, and impacts of adaptations were narratively synthesized in accordance with the Framework for Reporting Adaptations and Modifications to Evidence-Based Interventions. Results showed that most adaptations were proactive and focused on delivery methods, predominantly digitalization. While feasibility and acceptability were generally observed, the impacts of adapted programs were inconclusive. Inadequate reporting, especially regarding rationale, fidelity, facilitator capacity building, stakeholder involvement, and decision-making processes, was noted. The review recommends enhanced planning, documentation, and reporting of program adaptations using established guidelines, as well as process and impact evaluations.

Child maltreatment is an urgent public health and human rights issue. WHO (2022) defines child maltreatment as “all forms of violence against young people under 18 years old, whether perpetrated by parents or other caregivers” (p. 1). It is estimated that worldwide, more than 50% of children aged 0 to 19 experienced violence in the past year, with child maltreatment perpetrated by household members being the most common form (Devries et al., 2018). Ninety-three percent of the disability-adjusted life years lost due to violence against children, including child maltreatment, occur in low- and middle-income countries (LMICs; WHO, 2019). Child maltreatment is a known risk factor for a range of negative individual and societal outcomes, including death and injuries, mental and physical health problems, cognitive impairments, lower educational attainment, lower employment status, and substantial economic burden associated with human capital loss and remediation of the impacts of child maltreatment (X.Fang et al., 2015; Hughes et al., 2017; Teicher et al., 2016). It also leads to persistent involvement and intergenerational transmission of violence (Widom et al., 2015).

Parenting programs are a key strategy for reducing child maltreatment perpetrated by parents/caregivers (WHO, 2016, 2023). They are often grounded in social learning and attachment theories (Bandura, 1971; Bowlby, 1969) and can be delivered as universal, selective, or indicated interventions depending on the risk levels (O’Connell et al., 2009). Evidence shows that parenting interventions can modify risk (e.g., parental stress, harsh discipline, and child disruptive behaviors) and protective factors (e.g., positive parenting practices and positive parent–child interactions) related to child maltreatment, as well as reduce the actual incidence of child maltreatment (Barlow et al., 2006; Chen & Chan, 2016; Lundahl et al., 2006; Vlahovicova et al., 2017) across a range of global contexts (Knerr et al., 2013). Such programs are also effective among families of children with disabilities (Z.Fang, Barlow, & Zhang, 2022; Z.Fang et al., 2023).

COVID-19 has heightened children’s risk of experiencing child maltreatment. Stressors, such as social isolation, economic difficulties, an increase in family violence, and limited access to supportive services, have accumulated during COVID-19 to threaten child safety and well-being (Cappa & Jijon, 2021). For instance, a study examining data from 48 child helplines across 45 countries revealed a global increase in helpline contacts during the pandemic, with certain countries experiencing a notable rise in reports of violence (Petrowski et al., 2021). Despite the growing need for parenting interventions during the pandemic, restrictions on movement and social gatherings hindered the traditional provision of in-person parenting support. Consequently, there emerged an unprecedented need for in-person programs to swiftly transition to digital platforms for continued delivery. The swift paradigm shift necessitated considerable endeavors from program designers, implementation agencies, and dedicated staff. Various resources, exemplified by initiatives like the Rapid Response Virtual Home Visiting project in the United States, have also been expeditiously established to guide and support the adaptation process.

Implementation science is a multidisciplinary field dedicated to bridging the gap between research and practical application. It focuses on identifying effective strategies for fostering the adoption and sustainability of evidence-based interventions. Implementation outcomes, distinct from effectiveness outcomes, act as crucial prerequisites for achieving desired impacts. Key implementation outcomes include acceptability, feasibility, adaptation, and fidelity. Acceptability pertains to stakeholders’ perception that a given program is agreeable and satisfactory, and feasibility assesses the program’s successful delivery within a specific setting (Proctor et al., 2011). Adaptation involves modifying an intervention to better align with a new context (Moore et al., 2021), while fidelity refers to the degree to which an intervention adheres to the original protocol (Dusenbury et al. 2003), ensuring effective program functioning (Martin et al., 2021). Evaluation of implementation outcomes not only advances understanding of the implementation process but also facilitates the replication and transfer of interventions across diverse settings.

Notwithstanding the commendable efforts to address the unforeseen challenges posed by a global emergency, the move toward digital delivery has raised concern about the potential consequences on user experience and program impacts. The digitalization of interventions is an example of the tension between program adaptation and fidelity. For example, in the pandemic, an in-person parenting program may be adapted to be delivered through video-conferencing platforms for parents, an approach more feasible than meeting in-person to have discussions. However, this could make the program less effective by reducing social learning, which is a part of the intervention theory of change.

A range of frameworks and guidance, such as ADAPT(a guidance for intervention adaptations in new contexts, Moore et al., 2021) and FRAME (Framework for Reporting Adaptations to Evidence-Based Interventions; Stirman et al., 2019), have emerged to mitigate these challenges by promoting context-intervention fit. However, these frameworks are still gaining traction in the field of adaptation, and it remains unclear how they are being used in the context of parenting research.

There have been reviews on the adaptation of various health programs affected by the COVID-19 pandemic. For instance, Khurshid et al. (2020) conducted a rapid narrative review to identify adaptations made in healthcare quality improvement training and education in response to the pandemic. Similarly, Raphael et al. (2021) conducted a systematic review to synthesize service adaptations in mental health services during COVID-19 and other public health crises. Despite these efforts, there is a notable absence of systematic reviews examining the adaptations of parenting interventions during the pandemic.

In the field of parenting interventions, Breitenstein et al. (2014) carried out a systematic review to summarize the use of technology and digital delivery methods in parenting programs. Their review investigated the digital methods used, program completion rates, and reported outcomes, but it was conducted a decade ago and new data has emerged. More recently, Solís-Cordero et al. (2022) conducted a systematic review of the effectiveness of remotely delivered parenting programs on parent–child interaction and child development. However, their emphasis was primarily on quantitative studies and program effects, with limited reporting on implementation aspects. In addition, Klapow et al. (2024) undertook a systematic review examining the implementation feasibility and acceptability of parenting programs with a focus on those delivered via chatbot. Xie et al. (2023) also conducted a narrative systematic review on the modality and user experience of digital parenting interventions, specifically focusing on fathers of infants. Overall, there is a lack of comprehensive reviews synthesizing all parenting interventions adapted during the pandemic.

Given the impracticality and ethical concerns associated with conducting research during such a crisis, much of the research on these adaptations is underway. This evolving landscape presents a ripe opportunity for synthesis that offers insights into how and why these adaptations were undertaken, and to inform ongoing and future research on whether the adaptations made in response to COVID-19 are compromising the integrity of interventions or, conversely, contributing to their implementation and effectiveness.

This review focuses on parenting programs aiming to reduce physical and emotional child maltreatment perpetrated by primary caregivers, or relevant risk and protective factors. It aims to investigate how parenting interventions have been adapted to the COVID-19 pandemic and its sequelae. The review is guided by the FRAME framework (Stirman et al., 2019), which is recognized as one of the most comprehensive and up-to-date adaptation classification frameworks. This framework is designed to systematically document and report all essential facets of intervention adaptations. It aims to support all stakeholders involved in adaption to structure and systematically report the process. FRAME provides valuable insights into the rationale, nature, process, and impacts of intervention adaptations. Its application facilitates the understanding and advancement of intervention implementation and scale-up processes. Informed by FRAME, this review sought to answer the following research questions:

1. What was the rationale for adapting parenting interventions?
2. What types of adaptations were made?
3. What was the feasibility and acceptability of adaptation?
4. What were the intervention outcomes in child maltreatment and its risk and protective factors, assessed in experimental and descriptive studies, as well as reported qualitatively?

By examining the feasibility, acceptability, and potential impacts of programs adapted during the pandemic, this review can inform future adaptation of parenting interventions for digital delivery, as well as the design and delivery of digital or hybrid (combining in-person and digital) parenting interventions to reach families remotely.

## Methods

This review was guided by the Cochrane guidance for conducting systematic reviews (Higgins & Green, 2011) and followed the PRISMA guidelines, a set of evidence-based minimum items for reporting systematic reviews (Page et al., 2021). The results-based convergent synthesis design also was used to inform the review process (Noyes et al., 2019). To enhance transparency and minimize reporting bias, the review was pre-registered with PROSPERO (CRD42022330732), an international database aiming to provide a comprehensive register of systematic review protocols before they are conducted.

### Inclusion and Exclusion Criteria

Studies were included if they reported on parenting programs for parents or primary caregivers of children aged under 18. The interventions were included if they were designed based on social learning and attachment theories to increase parenting knowledge, change parental attitudes, and improve parenting skills, aiming to reduce emotional or physical child maltreatment or alter factors associated with child maltreatment (such as child behaviors, parental mental health, positive parenting, and parent–child relationships). Studies on interventions that focused on specific child safety issues (e.g., accident and injury) were excluded. To be included, interventions must have been developed prior to COVID-19 and been adapted for delivery during the pandemic and its sequelae. Studies utilizing any methodological approach and conducted in any context were eligible.

### Search and Screening

Seven international databases, three Chinese regional databases, and six gray literature repositories (see Supplemental Appendix 4—List of Databases and Grey Literature Repositories) were searched for studies published in English and Chinese between January 1, 2020 and December 1, 2022. Only articles published in English and Chinese were included due to the languages spoken by the reviewers. Reference lists of included studies were hand-searched for relevant reviews and articles, and any reviews identified during the search were examined for additional articles. The search strategy included terms related to parenting interventions, program adaptations, and the COVID-19 pandemic (see Supplemental Appendix 5—Sample Search Strategies). The screening of titles and abstracts was conducted using Rayyan. ZF, proficient in both English and Chinese, screened all titles and abstracts, and an additional quality check was performed by YS, who double-screened a randomly selected 10% of all English references. The results were highly consistent. Conflicts were resolved through discussion. ZF subsequently retrieved and assessed the full texts of all potentially eligible studies. The final list of included studies was confirmed with YS and RE.

### Data Extraction

Data extraction was informed by FRAME and the Template for Intervention Description and Replication (TIDieR) Checklist and extracted items were as follows: study information, study design, context, program features (program model, level of prevention, delivery modality, delivery method, intensity, location, and facilitator qualification), and participant characteristics; timing and rationale for adaptation, actors, types of adaptation, adaptations occurred at what level of delivery, nature of content modification, fidelity to core components, user experience, and impact of the adapted version; and for quantitative studies: measures and outcomes. We extracted both direct quotes and author reflections, presented in narrative or visualized forms. MM and LC each extracted 50% of the included studies, with all extractions verified by the third reviewer, ZF. Issues were resolved through discussion.

### Quality Appraisal

Quality assessment was conducted based on the Mixed Methods Appraisal Tool (MMAT; Hong et al., 2018), which allows for the assessment of studies with different methods using one appraisal tool. The MMAT consists of two screening questions to determine whether a paper is an empirical study, followed by five criteria for each type of design (i.e., qualitative study; randomized controlled trial [RCT]; non-RCT, in which people are allocated to different conditions using methods that are not random; quantitative descriptive study; and mixed-methods study). Criteria were rated “Yes,” “No,” or “Can’t Tell.” MM and LC each quality-appraised 50% of the included studies, with the third reviewer, ZF, verifying all decisions. Uncertainties were resolved through discussion with YS.

### Data Analysis and Synthesis

We drew on principles of the results-based convergent design, where qualitative and quantitative data are analyzed separately and then combined to address a research question (Noyes et al., 2019). First, we synthesized quantitative and qualitative aspects separately per research question. For the quantitative synthesis, given the limited number of quantitative studies and the diversity of study designs and outcome measures, we conducted a synthesis without meta-analysis using the Synthesis Without Meta-analysis (SWiM) guidelines, specifically designed to facilitate transparent reporting in reviews of interventions employing synthesis methods other than the meta-analysis of effect estimates (Campbell et al., 2020). For the qualitative synthesis, we performed framework syntheses of direct quotes and author reflections using ATLAS.ti, a specialized software developed for qualitative data analysis and designed to enhance the rigor and efficiency of the data analysis process (Carroll et al., 2011). A preliminary coding framework was developed using a subset of data. The remaining data were then coded and mapped against this framework. For data that did not fit into pre-existing themes, inductive thematic analysis (Braun & Clarke, 2006) was applied to generate new themes or revise existing themes. The process was iterated to ensure that the framework allowed for a comprehensive representation of the data. Second, where applicable, results from both syntheses were combined to answer specific research questions. Third, we cross-referenced adaptations with feasibility and outcomes by mapping research design and key findings against program modifications. All data can be made available upon request.

## Results

### Search Results

**Figure 1.**
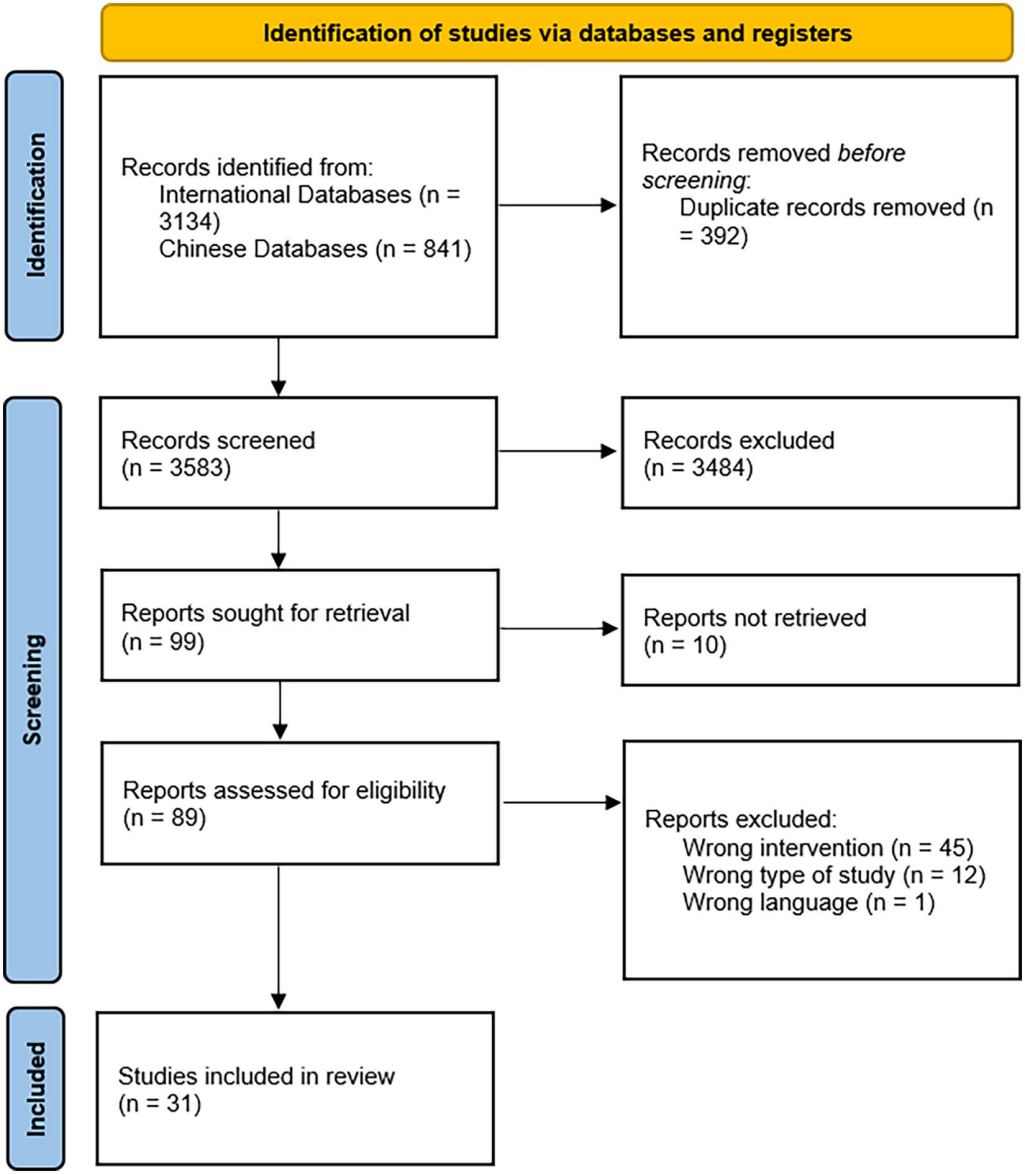
Flow chart.

We screened the titles and abstracts of 3,583 studies. Eighty-nine full texts were retrieved. Of these, 31 studies met the inclusion criteria (see Figure 1).

### Study Characteristics

#### Study Year and Country

Characteristics of the included studies are presented in Supplemental Appendix 1. The included studies were published between 2020 and 2022. Studies were conducted in six WHO regions: America (*n* = 20), Europe (*n* = 4), Southeast Asia (*n* = 4), Africa (*n* = 3), Eastern Mediterranean (*n* = 1), and West Pacific (*n* = 1). Twenty-four studies were conducted in high-income countries, with five in upper-middle-income countries, two in lower-middle-income countries, and three in low-income countries. Out of the 31 studies, 18 were conducted in the United States and 3 in China. One study was conducted in each of the following countries: Australia, El Salvador, France, Portugal, South Africa, the United Kingdom, and the United Arab Emirates. Three studies were conducted in multiple countries: du Toit et al. (2021) in Zambia, Tanzania, and Uganda; Franz et al. (2022) in the United States and South Africa; and Sherr et al. (2022) in the United Kingdom, United States, South Africa, Zimbabwe, Israel, Sri Lanka, Pakistan, and India.

#### Intervention and Participants

We used the TIDieR checklist for reporting program features (Hoffmann et al., 2014). Twenty-four models of parenting programs were identified (Supplemental Appendix 1), such as Parent–Child Interaction Therapy (*n* = 4), Parenting for Lifelong Health (*n* = 3), Attachment and Biobehavioral Catchup (*n* = 2), and Developing Our Children’s Skills (*n* = 2). Each study involved different populations and settings, even when using the same program model. Hence, we considered them as distinct programs. All programs sought to prevent child maltreatment, as classified using the US National Research Council classification of prevention program types (O’Connell et al., 2009). Five studies provided universal services with respect to child maltreatment prevention; 8 were selective for at-risk families; 17 were indicated focusing on families of children with substantial behavioral concerns, such as children diagnosed with attention-deficit/hyperactivity disorder, autism, and intellectual disabilities; and one provided both indicated and selective services to families at different risk levels.

Program facilitators were professionals in 18 studies, semi-professionals in 4, and laypersons in 2; the remaining studies provided no relevant information. Fifteen programs were group based, 11 were individual based, 5 offered both formats, and the remaining studies did not specify. Few studies reported program locations, with two in urban settings, one in rural areas, and one in both rural and urban contexts. Program length varied from 6 weeks to 22 months, with 6 to 24 sessions. The frequency with which program sessions were delivered ranged considerably, with 13 interventions intended to be weekly, 2 daily, and 1 twice per week. Twenty-seven studies involved male and female caregivers and three included only female caregivers. All studies involved a mixture of male and female children, except for one study focusing on a single girl. One study involved grandparents (Canário et al., 2021), and another involved teachers (McDevitt, 2021).

#### Study Design

Twenty studies were quantitative, including 4 RCTs; 4 non-RCTs, and 12 descriptive studies. Fourteen studies used a qualitative approach, of which 10 conducted individual interviews, focus group discussions, or open-ended survey questions; 3 utilized case studies; and 1 coded field notes. Six studies adopted a mixed-methods approach. Three studies were non-empirical author reflections.

#### Quantitative Outcomes Targeted by the Included Interventions

Seven outcome domains were assessed in the 20 quantitative studies. They were the incidence of child maltreatment (*n* = 1) and risk or protective factors for child maltreatment, including child behaviors (*n* = 8), child development (*n* = 2), parental belief in harsh parenting (*n* = 1), parental mental health (*n* = 8), parenting practice (*n* = 8), and parent–child interaction (*n* = 3). The outcomes were measured using a wide range of scales, which are presented in Supplemental Appendix 1.

#### Quality Assessment

The methodological quality of the 28 empirical studies was assessed. Out of these, 23 studies had a clear research question and appropriate data collection methods. In 13 out of the 14 qualitative studies, the qualitative approaches and data collection methods were deemed suitable for answering the research question. In these 13 qualitative studies, findings were assessed as being adequately derived from the data, and the interpretation of results was sufficiently substantiated by the data. Maurice et al. (2022) was potentially deemed inadequate due to the inclusion of only one brief case study. In addition, there was coherence between qualitative data sources, collection, analysis, and interpretation in the 13 qualitative studies.

Regarding the four RCTs, three (Amaral et al., 2022; du Toit et al., 2021; Macam et al., 2022) reported an appropriate randomization process. Two studies (Amaral et al., 2022; du Toit et al., 2021) demonstrated adequate baseline group comparability and participant adherence to the intervention, and they also reported complete outcome data. However, only one study (Amaral et al., 2022) reported blinding of outcome assessors.

In the case of the four non-RCTs, there was a lack of information on the selection process for the non-randomized treatment groups in two studies (Agazzi et al., 2021, 2022). All of them used appropriate outcome measures and accounted for confounders in their analysis. Two studies (Agazzi et al., 2021; Schein et al., 2023) reported complete outcome data. Schein (2023) also indicated that the intervention was administered as intended.

As for the 12 quantitative descriptive studies, all of them utilized appropriate outcome measures with standardized tools and employed suitable statistical analysis to address the research questions. Out of these, 11 studies had a sampling strategy relevant to their research questions. The study by Gerow et al. (2021), which aimed to evaluate the efficacy of the intervention, was rated as having a low-quality sampling strategy as it included only four families, thereby limiting the power to detect intervention effects. Among the 12 studies, 9 were rated as having low non-response bias, while 3 studies did not provide sufficient information to assess non-response bias.

Regarding the six mixed-methods studies, three (Baggett et al., 2021; Canário et al., 2021; Caron et al., 2022) demonstrated a well-justified rationale for the mixed-methods design. These three studies were rated as high quality on this dimension as they effectively integrated and interpreted the quantitative and qualitative components to address their research questions. The qualitative and quantitative components in these three studies also adhered to the quality criteria of each design, and any divergences or inconsistencies between quantitative and qualitative results were adequately addressed.

### Adaptations

Our reporting of adaptions was structured using FRAME (Stirman et al., 2019).

#### Rationale, Timing, and Decision-Maker

All included studies reported a rationale and timing for the adaptations made in response to the pandemic. The most common reasons for adaptations were COVID-19 restrictions and health risks. The study authors indicated that the adaptations made were in response to the need to increase program feasibility and engage participants during the pandemic. The majority (94%) of the included studies had the opportunity to strategize and plan for adaptations before initiating a new round of delivery, except for studies by Al Sehli et al. (2021) and Shenderovich et al. (2023) which were compelled to make changes during the delivery process, without prior planning.

Overall, studies did not make explicit who was responsible for decision-making regarding adaptations. Based on the description of the adaptation process and author information, researchers were likely involved in adaptations in seven (23%) studies (Agarwal et al., 2022; du Toit et al., 2021; Macam et al., 2022; McIntyre et al., 2022; Roben et al., 2022; Schein et al., 2023; Sherr et al., 2022) and facilitators were likely involved in four (13%) studies (Amaral et al., 2022; Maurice et al., 2022; Shenderovich et al., 2018; Sherr et al., 2022).

#### Types of Adaptations

There were 4 types of adaptation reported in the 31 studies made to suit program delivery during the pandemic; there were changes to program content, delivery modalities, facilitator training, and fidelity measurements (Table 1).

**Table 1.** Adaptations Made During COVID-19.

Theme	Subtheme
Content	
1. Added content	Added COVID-19 content
	Added behavioral management content
	Added individual support
2. Removed content	Only focused on more advanced child abilities
Delivery/context	
1. Changed delivery modalities	Transitioned to remote delivery
	Used spacious offline venues
	Combined in-person and digital delivery
	Sent materials via mail and email
2. Created new engagement strategies	Promoted online self-referral
	Offered technical and internet support
	Used new facilitation methods
	Changed group activities
3. Changed session format	Changed session length
	Changed the total number of sessions
	Changed session frequency
	Changed group size
Training and evaluation	
1. Changed capacity-building methods	Changed training modality and tools
	Provided ongoing support
2. Promoted and measured fidelity	Provided supervisors monitoring
	Completed fidelity checklists
	Created a detailed action plan

**Table 2.** Summary of Critical Findings.

• Adaptations made to parenting programs delivered during the COVID-19 pandemic primarily focused on changing delivery modalities, participant engagement strategies, and session formats. Adjustments to program content, fidelity measures, and facilitator training were less frequently reported. • Adapted parenting programs demonstrated feasibility and acceptability, revealing both opportunities and challenges in participant engagement with digital delivery. • The evaluations of the impacts of the adapted programs yielded mixed results. • Insufficient reporting of adaptations is evident, particularly concerning aspects such as the rationale behind adaptations, fidelity consistency, capacity building, stakeholder involvement, and the decision-making process.

**Table 3.** Implications for Practice, Policy, and Research.

Practice	• Practitioners should tailor interventions to align with the new context and document these adaptations for future reference. • Collaborative efforts between practitioners and researchers are encouraged to create adaptation plans and document program implementation. • Enhanced support should be provided to facilitators to ensure their proficiency in implementing adapted programs.
Policy	• Policies should support adaptation of evidence-based parenting interventions with local context-aligned digital or hybrid delivery methods, which appear to be feasible and offer potential advantages, such as wider reach.
Research	• Future research should adhere to established guidelines for conducting and reporting adaptations, such as ADAPT, FRAME, and FRAME-IS (Framework for Reporting Adaptations and Modifications to Evidence-based Implementation Strategies). • Future research should also delve beyond describing what is adapted to include more explicit information on the rationale, stakeholder involvement, decision-making processes, capacity building, and considerations of implementation fidelity. • More research on the implementation and impact of adapted programs and on adaptations undertaken in LMICs is needed.

*Note*. FRAME = Framework for Reporting Adaptations to Evidence-Based Interventions; LMICs = low- and middle-income countries.

##### Content

Six (19%) studies reported adding or removing content. Ferrara et al. (2022), Liu et al. (2021), and McDevitt (2021) added mental health support in response to COVID-19, whereas Ferrara et al. (2022) and Shenderovich et al. (2023) provided general COVID-19 guidance, such as recommendations for social distancing and handwashing. Corvin et al. (2021) enhanced individual case management. Franz et al. (2022), reporting on a program supporting autistic families, added a session on behavioral management and also reduced program content to focus only on improving abilities in older and more developmentally advanced children with autism.

##### Delivery

All studies reported adaptations to delivery methods. These adaptations were (a) changing delivery modalities, (b) creating new engagement strategies, and (c) modifying session formats.

All studies, except Baggett et al. (2021), (97%) reported converting to digital delivery to reach families remotely, such as videoconferencing, live-streamlining, pre-recorded videos, text messages, voice notes, emails, online self-learning materials, phone calls, radio programs, and social media posts (Supplemental Appendix 1). Parenting for Lifelong Health programs also used printed handouts, which condensed core program content into simple tip sheets (du Toit et al., 2021; Shenderovich et al., 2023; Sherr et al., 2022). The intervention team in Shenderovich et al. (2023) continued to conduct in-person sessions in spacious venues. Five studies used a hybrid approach, offering both in-person and digital support (Caron et al., 2022; Garcia et al., 2021; Lo et al., 2023; Shenderovich et al., 2023; Sherr et al., 2022).

Fourteen (45%) studies reported using new engagement strategies. For example, Baggett et al. (2021) increased online self-referrals. Six studies offered technical assistance, supporting families in installing and navigating software (Agarwal et al., 2022; Agazzi et al., 2021, 2022; Corvin et al., 2021; Lewis et al., 2022; McIntyre et al., 2022), and two provided digital devices and internet access (Corvin et al., 2021; du Toit et al., 2021). Flexible scheduling was used by Corvin et al. (2021) and Lewis et al. (2022) to accommodate competing family priorities. Two studies introduced new group facilitation techniques (e.g., regularly looking at the camera and scanning participant facial expressions) to promote caregiver engagement (Ferrara et al., 2022; Fogler et al., 2020). Seven studies adapted session activities, involving procedures to set up the virtual environment, alternations in discussion formats, and more online conversations with families following each session (Agazzi et al., 2021; Canário et al., 2021; Cook et al., 2021; du Toit et al., 2021; Fogler et al., 2020; Franz et al., 2022; Gerow et al., 2021).

Eight (26%) studies reported four strategies to adapt session formats—changing session lengths, reducing the number of sessions, using longer gaps between sessions, and adjusting group size. To give a few examples, Lewis et al. (2022) increased session lengths to reduce the total number of sessions, whereas Fogler et al. (2020) and Lo et al. (2023) shortened session lengths without changing the total number of sessions. Fogler et al. (2020) emphasized key messages during the shortened sessions, while Lo et al. (2023) did not report the impact of shorter sessions on the amount of caregiver support. Franz et al. (2022) reduced the total number of sessions to focus on a portion of the program’s content. Al Sehli et al. (2021) introduced longer gaps between sessions. Agarwal et al. (2022) and Shenderovich et al. (2023) shifted to smaller parent groups conducted either online or in-person, whereas du Toit et al. (2021) reported larger online groups.

##### Facilitator Training

Four (13%) studies adapted facilitator training and support. For instance, Shenderovich et al. (2023) transitioned from in-person to digital training, whereas Garcia et al. (2021) combined virtual group training with pre-recorded training videos and one-on-one consultation. Garcia et al. (2021) also developed a facilitator manual for virtual delivery. Three of the studies reported offering ongoing support for online delivery, including regular and on-demand supervision (Garcia et al., 2021), a co-therapy mode to pair new facilitators with experienced ones (Garcia et al., 2021), periodical team debriefs (Corvin et al., 2021; Garcia et al., 2021), and informal mutual support among facilitators (Ferrara et al., 2022).

##### Fidelity Measurement

Two (6%) studies developed new strategies to maintain or measure fidelity. McIntyre et al. (2022) had supervisors either observe all live sessions or watch recordings, and research staff attended each session to complete a fidelity checklist. Corvin et al. (2021) developed a new implementation protocol to promote adherence and conducted regular supervisor check-ins.

#### Feasibility and Acceptability

In this section, we present a summary of themes and subthemes from the synthesis of qualitative data, organized under three categories—perceived benefits of digital delivery, challenges of providing parenting support during COVID-19, and stakeholder suggestions for improvement. Example quotes and references are shown in Supplemental Appendix 2.

##### Perceived Benefits of Digital Delivery

Seven (23%) studies reported on the benefits of digital delivery perceived by caregivers and program facilitators. Studies indicated that caregiver engagement had increased during and following COVID-19, which was thought to be due to fewer logistical barriers (e.g., COVID-19 restrictions, travel distance, and childcare), more opportunities to reinforce key messages, and greater comfort with participating online. Digital home visits were perceived to allow facilitators to reach more fathers and potentially improve caregiver learning by providing opportunities for learning in a natural environment, solving problems independently, and receiving flexible support. Facilitators also viewed digital delivery as promoting their professional growth by prompting them to rethink the program. Moreover, facilitators highlighted that digital delivery helped parenting programs adjust to the “new normal,” with technology referred to as central to program sustainability during and post-COVID-19.

##### Perceived Challenges of Digital Delivery

Six (23%) studies reported challenges according to facilitator perspectives regarding the provision of regular services. Technical and resource issues—such as the lack of devices, unreliable internet access, and lack of technological readiness—were commonly mentioned as the biggest challenges in providing remote support. Facilitators also mentioned privacy and online safety as major concerns. Digital delivery was also perceived to hinder the identification of child maltreatment since facilitators might only see what caregivers preferred to present. In addition, some facilitators found it difficult to track behavioral changes in parents, as it took additional time for caregivers to complete the questionnaire remotely.

These six studies also reported facilitator perspectives on barriers to engaging participants via digital delivery. Such barriers included difficulties in remote communication, lack of a structured setting, more distractions, and limited acceptability of remote programs. Difficulties in explaining concepts remotely, building strong relationships with families, and observing caregivers and their surroundings were perceived to contribute to ineffective communication. Facilitators also felt that they were unable to create an appropriate learning environment at times, as they had limited control of the space, and caregivers were reluctant to rearrange the home settings. Moreover, facilitators observed that caregivers had more distractions when attending remotely, such as due to the presence of children and pets at home, shifting family priorities, and an overload of stress and responsibilities (e.g., financial crisis). Facilitators also articulated that caregivers tended to view digital programs as less formal and were therefore less committed.

##### Stakeholder Suggestions for Improvement

Four (13%) studies reported on caregiver and facilitator suggestions for improvement on future adaptations. Caregivers highlighted the need for smaller group sizes and add-on in-person elements. Facilitators expressed the need for setting up boundaries with caregivers and receiving support from other facilitators, organizational leadership, and the wider network of family service providers.

#### Potential Impacts

This section summarizes the quantitative and qualitative information on program impacts. Supplemental Appendix 1 presents the quantitative measures of the program’s effect and process. Supplemental Appendix 2 presents the themes of stakeholder-perceived intervention impacts.

##### Child Maltreatment

Only one (3%) quantitative study (Amaral et al., 2022) found no significant difference between the digitalized program and the control in reducing the incidence of physical child maltreatment post-intervention. The control group appears to have received treatment-as-usual (no additional services). Based on caregiver gender, the study found that among female caregivers there was a reduction in reported physical violence.

##### Child-Level Associated Factors

Seven (23%) studies reported on child behavior problems. Among them, three quantitative descriptive studies reported fewer child behavior problems post-tests compared to baseline (Canário et al., 2021; Garcia et al., 2021; Gerow et al., 2021). Four studies with randomized or quasi-experimental control groups identified similar effects of the digitalized programs delivered using video-conferencing on reducing child behavior problems compared to an in-person program (Agazzi et al., 2021, 2022), waitlist control with treatment-as-usual until the follow-up data collection (du Toit et al., 2021), and treatment-as-usual (Amaral et al., 2022). Three (10%) studies reported on child development, with one quantitative descriptive study showing reduced body mass index (Canário et al., 2021), and an RCT finding no effects of the digitalized program delivered using video-conferencing in promoting child social, emotional, or language development, compared to waitlist control (du Toit et al., 2021). However, caregivers in the qualitative study of Lo et al. (2023) perceived that children had better social communication skills after the program.

##### Parent-Level Factors

Eight (26%) studies reported on parental mental health and eight (26%) reported on parenting practices. An RCT with waitlist control (du Toit et al., 2021), a non-RCT with treatment-as-usual (Liu et al., 2021), and two quantitative descriptive studies (Garcia et al., 2021; Traube et al., 2022) reported improved parental psychological functioning. In the qualitative interviews, caregivers also described improved parental mental health due to enhanced stress management and self-care skills, as well as increased social support (du Toit et al., 2021; Sherr et al., 2022). Two non-randomized controlled studies found no difference between the digitalized programs, compared to in-person programs, in terms of parental psychological functioning, with both online and in-person participants reporting decreases in stress, compared to baseline (Agazzi et al., 2021, 2022). In these two studies, no group did not receive a parenting intervention. One RCT found that the digitalized program exacerbated mental health distress, particularly stress among male caregivers, compared to treatment-as-usual (Amaral et al., 2022).

As to parenting practices, an RCT comparing a digitalized program with waitlist control (du Toit et al., 2021) reported the intervention group had better responsive parenting post-test. Three quantitative descriptive studies (Canário et al., 2021; Garcia et al., 2021; Lewis et al., 2022) reported higher levels of responsive and positive parenting post-test, compared to baseline for the same group. Yet, treatment effects were not detected in an RCT comparing delivery via videoconferencing to treatment-as-usual that involved no intervention (Amaral et al., 2022). One quasi-experiment compared videoconferencing to hybrid delivery, finding that both had similar outcomes (Schein et al., 2023). Specifically, the quasi-experiment found that, using observational measures of parental sensitivity, both videoconferencing and hybrid delivery of the program, which was initially solely delivered in person, demonstrated moderate effect sizes in improving parenting practices from pre- to post-intervention (Schein et al., 2023).

Caregivers in qualitative interviews also referenced increased parental self-efficacy, characterized by being more sensitive to child needs, having better understanding of child development, using less harsh discipline, and employing more positive parenting practices (Cook et al., 2021; du Toit et al., 2021; Sherr et al., 2022). As to parental attitude toward harsh parenting, du Toit et al. (2021) found no significant difference between the online parent groups and waitlist control in parental beliefs about physical punishment. Male engagement in caregiving was perceived to have improved in Ferrara et al. (2022).

##### Parent–Child Interaction

Five (16%) studies reported on parent–child interaction. Amaral et al. (2022) found that, compared to treatment-as-usual, the online parenting program did not significantly impact mother–child interactions, yet it had a significant negative impact on father–child interactions. In one case study (Melo et al., 2021) and several qualitative interviews with parents (du Toit et al., 2021; Lo et al., 2023; Sherr et al., 2022), caregivers perceived improvements in parent–child relationships.

### Cross-Referencing

Due to the diversity of research designs and adaptations, the limited number of studies, and the varying quality of the included studies, the cross-referencing did not identify clear patterns regarding which adaptations might correlate with greater feasibility or more favorable outcomes. Supplemental Appendix 3—Cross-Referencing Tables present the mapping results.

## Discussion

This global systematic review provides an overview of studies examining adaptations to parenting programs made during the COVID-19 pandemic. It aims to investigate why and how the programs have been adapted, as well as the feasibility, acceptability, and potential impacts of the adapted programs. It is hoped that the information and insights provided by this review will facilitate dialogue about digital adaptations to parenting programs.

### Findings

We identified 31 studies of parenting programs adapted due to COVID-19, involving both male and female caregivers and children with different clinical conditions. We found that the adaptations were predominantly proactive and aimed to both reduce the health risks and increase program feasibility and participant engagement. Adaptations were made to program content, delivery, facilitator training, and fidelity measurements, with most adaptations made to delivery methods and transitions from in-person programs to digital or hybrid delivery. Unlike previous findings that indicated digital parenting interventions were primarily delivered via websites or web-based portals (Xie et al., 2023), our review identified over 10 types of delivery modalities, such as videoconferencing, text messages, phone calls, and radio broadcasting. This demonstrates that the pandemic has expedited the diversification of parenting service provision (Cluver et al., 2020). A range of new engagement strategies were therefore created, such as offering technical support and using online group facilitation techniques and new session formats to improve the implementation of digital programs. A few studies reported making changes to the program content while several studies reported shifting formats of facilitator capacity building and fidelity monitoring.

Similar to a review finding that chatbot-led parenting interventions primarily delivered in high-income countries exhibited high retention rates and good acceptability (Klapow et al., 2024), our review also showed that the adapted programs demonstrated general feasibility and acceptability. We found that digital delivery could alleviate common logistic barriers to participation, a trend consistent with previous research indicating higher participation rates in digital programs compared to in-person versions (Perrino et al., 2018). This also aligns with findings from a previous review of home visiting programs that transitioned to virtual delivery during the pandemic, which reported comparable service indicators, such as caseloads and completion rates, to pre-pandemic levels in some programs (Roben & Costello, 2022). The review also found that digital delivery promoted caregiver learning and interaction and increased the reach of male caregivers. This echoes findings from a previous review of digital parenting interventions for fathers of infants, which found that these interventions were deemed to increase support between partners in childcare and improve paternal confidence in parenting (Xie et al., 2023). A review on virtual adaptation of healthcare quality improvement training during COVID-19 also found that online training programs increase program reach and promote participant learning by enhancing flexibility and participant control. Furthermore, our review highlighted that the use of digital technology was deemed as key to program sustainability post-pandemic. However, digitalization is not a straightforward undertaking. Despite the reported benefits, participant engagement remained an ongoing challenge (Butler et al., 2020). Similar to other studies (O’Neill et al., 2020; Racine et al., 2020; Sanders et al., 2012), our findings showed that the benefits of promoting participation might be compromised by technical problems, resource constraints, facilitator inexperience in digital delivery, difficulties in remote teaching, and less caregiver commitment which all could result in high attrition and inequitable participation, excluding families with least digital resources. Overall, our results align with a previous review of digital parenting interventions for fathers of infants, which found mixed findings regarding program feasibility and acceptability (Xie et al., 2023).

To improve participant experiences within the digital programs, the review findings suggest that caregivers need more personalized experiences and interactions with facilitators, which is in line with previous studies showing the importance of individualized parenting support ( Z.Fang, Lachman, et al., 2022) and human elements in digital health programs to tackle individual challenges (Harris et al., 2020). Facilitators also reported the need for wider support from peers and organizations and clear boundaries with participants.

Previous reviews have shown that digital programs have comparable effectiveness to in-person programs and, compared to no treatment, can help improve child behaviors, parenting style, parental mental health, and parent–child interactions (Baumel et al., 2016; Florean et al., 2020; Spencer et al., 2020). However, this review found mixed results, with some quantitative studies finding positive impacts for the digital adaptations, some studies, including the RCTs, finding null effects, and one study suggesting some potential negative impacts. These findings align with a systematic review that investigated the effectiveness of remotely delivered parenting programs in enhancing parent–child interaction and child development. The review similarly encountered mixed results and insufficient evidence to conclude (Solís-Cordero et al., 2022). It should be noted that we also included a range of study designs with a limited number of studies using a controlled trial, making it difficult to draw a definite conclusion. Several controlled studies lacked clear information on the control conditions. We identified only one RCT that reported positive impacts compared to a waitlist control, and one RCT with negative impacts. One possible explanation for the lack of effects in other RCTs could be difficulties with participant engagement, as reported in the qualitative synthesis.

### Evidence Gaps

Regardless of the challenges of the pandemic, a number of parenting programs were adapted and delivered, and we identified a range of studies examining these adaptations. However, there were several evidence gaps. Six studies reported content adaptations, but there was a general lack of explicit justification for altering program content. Despite the modifications to program content and delivery, only four studies reported corresponding responses made to facilitator capacity building to equip facilitators with the skills necessary to deliver interventions in new contexts. Furthermore, we found it challenging to code the degree to which programs were delivered with fidelity to their original models as only a few studies described strategies to measure implementation fidelity. The adaptations were made proactively in all but two studies, which might have reduced the risk of adaptations undermining program mechanisms of change. However, proactive adaptations do not guarantee adherence nor do they represent what actually occurred in the field where facilitators consistently face new challenges and complex family needs that make comprehensive proactive planning difficult and put fidelity at risk (Shenderovich et al., 2023). To understand the process of adaptation, FRAME suggests recording the level at which adaptations occur—for instance, for whom (i.e., an individual recipient, a specific intervention cohort, or a particular population) the adaptations were made and by whom (i.e., a facilitator, a unit within an organization, the entire organization, or the entire service network; Stirman et al., 2019). However, such information was not often explicit in the included studies. This inhibited our understanding of the power dynamics between different stakeholders during the adaptations and their potential relationships with program impacts. In the impact evaluations, information on study design was sometimes missing. Finally, the evidence we gathered was largely from high-income countries which limits our understanding of parenting program adaptation in LMICs.

### Strengths

This review provides an important contribution to the literature by synthesizing how parenting programs were adapted to suit the demands presented by the COVID-19 pandemic. The included studies involved a diverse group of male and female caregivers encompassing various ethnicities (such as White, Asian, African, Hispanic, and Arabic, as well as those of mixed heritage). The studies also reflected parenting interventions delivered in all six WHO regions, covering a mixture of high-, middle-, and low-income countries. The participating families involved families of children with and without disabilities.

### Limitations

A limitation of this review is that many studies were not able to keep records of all of the adaptations made to programs during the pandemic due to limited resources and heightened pressures. As a result, the present review cannot provide a full picture of the adaptations made by parenting programs during the pandemic. Along these lines, studies reporting on adaptations may have reported on programs that were well-resourced and better implemented than other programs, leading to publication bias. As a result, the findings may have limited generalizability to low-resource settings wherein other adaptations may have been required to suit these contexts. In addition, many studies did not report on the socioeconomic status of parent participants so the extent to which programs reached these groups is not known. Limited process evaluation information on the quality of delivery was available. The review was also limited to studies published in English and Chinese languages, resulting in the omission of relevant studies published in other languages. We only conducted double screening on 10% of the English references, deviating from the Cochrane guidelines and potentially introducing selection bias. It was also not possible to conduct double screening for Chinese references because only one author was proficient in Chinese.

### Conclusion

This review provides insights into the adaptations of parenting interventions during the COVID-19 pandemic and its aftermath. By synthesizing a diverse range of studies, we have shed light on the strategies employed to maintain the delivery of parenting programs when in-person sessions were inhibited and to reach families remotely using digital methods.

Our findings underscore the critical need to adapt evidence-based interventions in response to unprecedented situations. The shift to digitalization presented both benefits and challenges. Overall, the adaptations implemented during the pandemic were found to be feasible and acceptable, as reported in various studies. In addition, fidelity to the original program designs could be maintained through online training. This suggests that the adaptations made during the pandemic can be both practical and acceptable across different types of parenting programs. However, while the adapted digital programs generally demonstrated feasibility and acceptability based on stakeholder perspectives, the varied outcomes in terms of their potential impacts highlight the complex interplay between adaptation and fidelity.

We also identified a comprehensive list of adaptations reported for studies focusing on parenting interventions aimed at improving parenting practices and addressing child maltreatment. This mapping provides valuable guidance on program options for mitigating child maltreatment and addressing its risk and protective factors during crises or transitions to digital formats.

### Implications

The findings of this review may have implications for practice, policy, and research. For practitioners, the review suggests that it is important to align interventions with the evolving context as well as to document their experiences to inform future practices. Resources, such as support with digital access, are necessary for high-quality and equitable digital delivery. Collaborative efforts between practitioners and researchers may prove to be valuable in formulating adaptation plans and documenting implementation strategies. Furthermore, the review suggests that facilitators should be adequately supported by peers, organizations, and the wider service network in implementing adapted and digital programs. From a policy perspective, this review suggests that policies supporting the adaptation, evaluation, and scaling of evidence-based parenting interventions with context-aligned digital or hybrid delivery methods hold promise. The pandemic has unveiled the potential of digital interventions, providing an opportunity to leverage this momentum to further enable the adoption of technology-enhanced parenting interventions that are tailored to local needs.

However, further impact and process evaluation are needed to establish the processes and effects of digital delivery of programs. Future adaptation studies should use established frameworks such as ADAPT (Moore et al., 2021) for conducting adaptations along with FRAME (Stirman et al., 2019) or FRAME-IS (Miller et al., 2021) for comprehensive documentation of adaptations. Research should delve deeper into the rationale behind adaptations, stakeholder engagement, decision-making processes, facilitator capacity building, and the maintenance of implementation fidelity. In addition, more research specifically focusing on adaptations in LMICs is needed.

## Supplemental Material

sj-docx-1-tva-10.1177_15248380241266183 – Supplemental material for Parenting Interventions During the COVID-19 Pandemic: A Systematic Review of the Rationales, Process, Feasibility, Acceptability, and Impacts of Adaptation Supplemental material, sj-docx-1-tva-10.1177_15248380241266183 for Parenting Interventions During the COVID-19 Pandemic: A Systematic Review of the Rationales, Process, Feasibility, Acceptability, and Impacts of Adaptation by Zuyi Fang, Mackenzie Martin, Lauren Copeland, Rhiannon Evans and Yulia Shenderovich in Trauma, Violence, & Abuse
