# Supplementary material for: Parenting Interventions During the COVID-19 Pandemic: A Systematic Review of the Rationales, Process, Feasibility, Acceptability, and Impacts of Adaptation
Source: Trauma Violence Abuse. 2024 Jul 31;25(5):3887–902. doi: 10.1177/15248380241266183 (PMC11545144; doi:10.1177/15248380241266183)
Supplement: sj-docx-1-tva-10.1177_15248380241266183 – Supplemental material for Parenting Interventions During the COVID-19 Pandemic: A Systematic Review of the Rationales, Process, Feasibility, Acceptability, and Impacts of Adaptation [file sj-docx-1-tva-10.1177_15248380241266183.docx]

**Appendices**

Table of Contents

[Appendix 1 Characteristics of Included Studies Table 1](#_Toc159248226)

[Appendix 2 Framework Synthesis of Qualitative Data: Themes and Example Quotes 9](#_Toc159248227)

[Appendix 3 Cross-Referencing Tables 16](#_Toc159248228)

[Appendix 4 List of Databases and Grey Literature Repositories 34](#_Toc159248229)

[Appendix 5 Sample Search Strategies 34](#_Toc159248230)

# Appendix 1 Characteristics of Included Studies Table

| **Study ID** | **Country** | **Design & Data Types** | **Program** | **Type** | **Target Population** |
| --- | --- | --- | --- | --- | --- |
| **Agarwal 2022** | USA | non-empirical (author reflections) | Child–Parent Relationship Therapy | selective | children with specific mental health or behavioural issues |
| **Agazzi 2022** | USA | quantitative: non-randomized controlled trial (control: in-person version) | Developing Our Children’s Skills K-5 (i-DOCS K-5) | selective | children who were perceived to have disruptive behaviours, no severity score or diagnosis required |
| **Agazzi 2021** | USA | quantitative: non-randomized controlled trial (control: in-person version) | internet-Helping Our Toddlers, Developing Our Children’s Skills (i-HOT DOCS) | selective | children who have challenging behaviours |
| **Al Sehli 2021** | United Arab Emirates | qualitative: case study | Parent-Child Interaction Therapy | selective | children with ADHD or epilepsy |
| **Amaral 2022** | El Salvador | quantitative: RCT (control: treatment-as-usual) | a stress management and positive parenting techniques program | universal | all families |
| **Baggett 2021** | USA | quantitative: descriptive (number of referrals) | Mom and Baby Net & Depression and Developmental Awareness | indicated | infants who were at elevated risk for poor social emotional and communication development as a function of maternal depression and adverse mother-infant interactions that exacerbate the detrimental effects of poverty |
| **Barnett 2021** | USA | quantitative: descriptive (post-test) qualitative: open-ended questions | Internet-Delivered Parent-Child Interaction Therapy | universal | all families |
| **Canário 2021** | Portugal | quantitative: descriptive (pre-post) qualitative: semi-structured interviews | Group Triple P | selective | overweight or obese children |
| **Caron 2021** | USA | quantitative: descriptive (fidelity scores) | Attachment and Biobehavioural Catchup - Infant and Toddler versions | indicated | infants and toddlers who have experienced early adversity |
| **Cook 2021** | Australia | qualitative: semi-structured interviews | Circle of Security-Parenting | indicated | women and their families experiencing moderate to severe mental illness during pregnancy and up to 12 months postpartum |
| **Corvin 2021** | USA | non-empirical (author reflections) | Positive Parenting Partnership | indicated and selective | families at risk of CAN and families involved in CAN services |
| **du Toit 2021** | Zambia, Tanzania, Uganda | quantitative: RCT (control: waitlist) qualitative: semi-structured interviews | Parenting for Lifelong Health - Sharing Stories | universal | all families |
| **Ferrara 2022** | USA | qualitative: focus group discussions | Army New Parent Support Program | indicated | military families who face unique challenges such as geographic relocations and potential physical and mental health problems due to combat exposure. |
| **Fogler 2020** | USA | qualitative: open-ended survey questions and focus group discussions, | Bootcamp for Attention-Deficit/Hyperactivity Disorder | selective | children who were recently diagnosed with ADHD |
| **Franz 2022** | USA, South Africa | non-empirical (author reflections) | Early Start Denver Model–Informed Caregiver Coaching | selective | children with autism |
| **Garcia 2021** | USA | quantitative: descriptive (pre-post) | Internet-Delivered Parent-Child Interaction Therapy | selective | children with elevated child disruptive behavior |
| **Gerow 2021** | USA | quantitative: descriptive (concurrent multiple-baseline) | a mixture of self-directed and on-one-one parent coaching | selective | children with autism |
| **Lewis 2022** | UK | quantitative: descriptive (pre-post) | Enfys Nurturing Attachments Groups | selective | children and young people who had been exposed to developmental trauma, most of whom were looked after. |
| **Liu 2021** | China | quantitative: non-randomized controlled trial (control: treatment-as-usual) | WeChat-Based Parenting Training | selective | children with autism |
| **Lo 2022** | China | quantitative: descriptive (post-test) qualitative: focus group discussions | Multi-Family Group model | selective | children with ID |
| **Macam 2022** | USA | quantitative: RCT (control: waitlist) qualitative: field notes | Incredible Years Basic Parent Training Program | universal | all families |
| **Maurice 2021** | France | qualitative: case study | Behavioral Parent Training | selective | children with ADHD |
| **McDevitt 2021** | China | qualitative: semi-structured interviews | Parent Education and Training Program | selective | children with autism |
| **McIntyre 2022** | USA | quantitative: descriptive (post-test) | Behavioral Parent Training | selective | children with developmental delay |
| **Melo 2021** | USA | quantitative: descriptive (pre-post) qualitative: case study | Parent-Child Interaction Therapy | selective | children with ADHD |
| **Roben 2022** | USA | quantitative: descriptive (process measures) | Attachment and Biobehavioral Catchup | indicated | infants and toddlers who have experienced early adversity |
| **Schein 2022** | USA | quantitative: non-randomized controlled trial (control: hybrid delivery) | Attachment and Biobehavioral Catchup | indicated | infants and toddlers who have experienced early adversity |
| **Shenderovich 2022** | South Africa | qualitative: semi-structured interviews | Parenting for Lifelong Health for Young Children and for Parents and Teens | indicated | disadvantaged families |
| **Sherr 2022** | UK, USA, South Africa, Zimbabwe, Israel, Sri Lanka, Pakistan, and India | qualitative: semi-structured interviews & open-ended questions | Parenting for Lifelong Health COVID-19 Resources | universal | all families |
| **Traube 2022** | USA | quantitative: descriptive (cross-sectional survey) | virtual early childhood home visitation services | indicated | families covered by safety net |
| **Yi 2021** | USA | quantitative: RCT (the same intervention but with modifications during the onboarding meeting and progress monitoring) | Telehealth Applied Behavior Analysis Parent Training | selective | children with autism |

**(continued)**

| **Study ID** | **Facilitator** | **Format** | **Duration** | **Session Number** | **Modality** | **Outcomes and Measures** |
| --- | --- | --- | --- | --- | --- | --- |
| **Agarwal 2022** | professional | group | 10 weeks | 10 | videoconferencing, pre-recorded videos |  |
| **Agazzi 2022** | professional | group |  | 6 | videoconferencing | child behavior: SDQ, ECBI parental mental health: PHQ-9, DOCS Parenting Stress Measure |
| **Agazzi 2021** | professional | group |  | 6 | videoconferencing | child behavior: ECBI parental mental health: DOCS Parenting Stress Measure |
| **Al Sehli 2021** | professional | individual | 8-14 months | 17 to 25 | virtual delivery |  |
| **Amaral 2022** |  | individual | 2 months | weekly texts | text messages/voice notes/emails | child behavior: World Bank Survey parental mental health: DASS-21 parenting practice: Barratt Impulsiveness Scale violence against children: Prevention of Child Abuse and Neglect Screening Tool, Parent Version parent-child interaction: Family Care Indicators instrument |
| **Baggett 2021** |  | group | 32 months | 14 |  | descriptive mapping of referrals before and during covid |
| **Barnett 2021** | professional | individual |  |  | videoconferencing | facilitator work status |
| **Canário 2021** |  | mixed | 17 weeks | 14 group sessions; 4 individual sessions | videoconferencing | child behavior: SDQ, Lifestyle Behaviour Checklist child development: BMI z-score parenting practice: Parenting Scale |
| **Caron 2021** | professional | group |  |  | a hybrid of in-person and digital delivery |  |
| **Cook 2021** | professional | group | 8 weeks |  | videoconferencing |  |
| **Corvin 2021** |  | mixed |  |  |  |  |
| **du Toit 2021** | layperson | group | 6 weeks |  | pre-recorded videos, text messages/voice notes/emails, online self-learning materials | child behaviors: SDQ, CBCL child development: Caregiver Reported Early Development Instrument parental attitude: Parent-Child Conflict Tactics scales parental mental health: PHQ-9, GAD-7, PSS parenting practice: Family Care Index |
| **Ferrara 2022** | professional | individual |  |  | videoconferencing, text messages/voice notes/emails, phone calls |  |
| **Fogler 2020** |  | group |  |  | videoconferencing |  |
| **Franz 2022** |  | group | 8 weeks in US; 12 weeks in SA | 8 or 12 | videoconferencing, pre-recorded videos, text messages/voice notes/emails, phone calls |  |
| **Garcia 2021** | professional | individual | 18 weeks |  | videoconferencing, pre-recorded videos, in-person delivery | child behaviors: ECBI, Child Compliance, BASC‑3 Internalizing Problems Composite Scores parental mental health: PSI-SF parenting practice: Positive “Do” Skills, Negative “Don’t” Skills parent-child interaction: DPCICS-IV |
| **Gerow 2021** | professional | individual | 6 or more weeks |  | videoconferencing |  |
| **Lewis 2022** | professional | group | 12 hours | varies | videoconferencing, pre-recorded videos | parental self-efficacy: brief parental self-efficacy scale, carer questionnaire, parental reflective functioning questionnaire |
| **Liu 2021** | professional | group | 12 weeks | 24 | live streamlining | parental mental health: self-rating anxiety and depression scales, PSI-SF, Herth Hope Index |
| **Lo 2022** | professional | group | 30 hours | 10 | videoconferencing, in-person delivery | satisfaction: Client Satisfaction Questionnaire |
| **Macam 2022** | professional | group | 12 weeks | 12 | videoconferencing | child behavior: CBCL parental mental health: PSI-SF, Epidemic–Pandemic Impacts Inventory parenting practice: Parenting Practices Inventory |
| **Maurice 2021** | professional | group | 10 days |  | videoconferencing |  |
| **McDevitt 2021** | semi-professional | mixed | 12 weeks |  | videoconferencing |  |
| **McIntyre 2022** | professional | mixed | 16 week | 16 | videoconferencing |  |
| **Melo 2021** |  | individual |  |  | videoconferencing | child behavior: ECBI parent-child interaction: DPCICSC26 |
| **Roben 2022** | semi-professional | individual | 10 sessions | 10 | videoconferencing |  |
| **Schein 2022** | semi-professional | individual | 10 sessions | 10 | videoconferencing | parenting practice: National Institute of Child Health and Development Observational Recording of the Caregiving Environment |
| **Shenderovich 2022** | semi-professional or layperson | group | 2-3 months | 10 to 12 | videoconferencing, printed handouts, in-person sessions |  |
| **Sherr 2022** |  | individual |  |  | pre-recorded videos, text messages/voice notes/emails, online self-learning materials, phone calls, radio, social media posts, in-person delivery |  |
| **Traube 2022** | professional | individual |  |  |  | parental mental health: PHQ-9, GAD |
| **Yi 2021** | professional | mixed | 60 days | 12 |  |  |
| Note: SDQ-Strengths and Difficulties Questionnaire; ECBI-Eyberg Child Behavior Inventory; CBCL-Child Behavior Checklist; PHQ-9-Patient Health Questionnaire-9; DASS-21-Depression, Anxiety, and Stress Scale; GAD-7-Generalized Anxiety Disorder-7; PSS-Parental Stress Scale; DPCICS-IV-Dyadic Parent–Child Interaction Coding System, Fourth Edition; PSI-SF-Parenting Stress Index-Short Form. | | | | | | |

# Appendix 2 Framework Synthesis of Qualitative Data: Themes and Example Quotes

| **Theme** | **Subtheme** | **Example Quote** |
| --- | --- | --- |
| **Perceived Changes Related to Individual and Familial Outcomes After the Programs**  **Studies that contribute to this theme:** (Cook, Bragg, & Reay, 2021; du Toit et al., 2021; Ferrara, Kaye, Abram-Erby, Gernon, & Perkins, 2022; Lo, Ma, Wong, & Yau-Ng, 2022; Sherr et al., 2022) | | |
| Better child development | \ | “My daughter was able to give a presentation about herself in front of so many people for 10 minutes. That was remarkable. I think my daughter was encouraged to try because she saw that someone of the same age as her had done it.” (Lo et al., 2022) |
| Increased parental self-efficacy | being more sensitive to child needs | “I can meet that need. It is quite simple. He just needs a cuddle.” (Cook et al., 2021) |
|  | better understanding of child development | “I have learnt that my child loves to see me which I didn’t observe at first and also that children are wise even if they can’t talk but they know many things” “The child is also a human and understands everything that is going on, although we as caregivers often feel that the child does not understand what is going on around them, so I think as parents we should not take business as usual thinking that children have static schedules such as bath, eat, sleep, and change diapers. But also, the child needs to do other activities like have fun, play and learn things as it nurtures their mind.” (du Toit et al., 2021) |
|  | less harsh discipline | “No more stress, hitting and spanking my children because of use of the tips.” (Sherr et al., 2022) |
|  | more positive parenting | “I now enjoy my children and plan activities with them.” “All tips for positive parenting were welcome. In times of crisis, somehow it is easier to get out of control and forget even those good parenting skills that we already have. What was new I certainly tried to change. Many tools calmed the domestic situation.”(Sherr et al., 2022) |
| Improved parental mental health | more stress management and self-care skills | “It has helped me a lot, to overcome my stress and to be kind to myself and to my loved ones, people around me, my child as well as other children. I have also learnt to be patient with a lot of children not only my child.”  “I'm now feeling better and I know how to manage and control my thoughts when I feel worried or stressed out, because I know it normal and sometimes when I feel that I opt to relax and sometimes seek for help from people who are very close to me and trustworthy” (du Toit et al., 2021) |
|  | more social support | “The programme has really helped me, especially with stress for example sometimes I would be upset for no reason and be moody but every time I would join the group chat and hear the stories of other parents how to be with children, how to cope with other people in the surrounding. Even if I was upset, I would cheer up.”  “I really liked this project especially when I met with other parents in the WhatsApp group it became like my other family, because we were sharing out our experiences in the upbringing of our children as we are learning from each other.” (du Toit et al., 2021) |
| Improved parent-child interactions | \ | “I spend a lot of time with them. I listen to them carefully. We discuss anything in a deep and subtle way.” (Sherr et al., 2022) |
| More male engagement in caregiving | \ | “My child is now free and happy to be with me, she doesn’t fear me anymore...she is no longer afraid of me, she feels happy when she sees me and prefer to ask me to show her digital books and asks me questions from that. Yes. I'm now better, I'm kind to my child, treating them well, politely and with love.” (male caregiver)  “There were some challenges because she could not accept me to carry her, I also tried to ignore her but when the project started I started carrying her, telling her stories and showing her the picture books you were sending. So this time the relationship is good, she’s responding very well” (male caregiver) (du Toit et al., 2021) |
| **Benefits of Digital Delivery**  **Studies that contribute to this theme:** (Barnett et al., 2021; Cook et al., 2021; du Toit et al., 2021; Ferrara et al., 2022; Lo et al., 2022; McDevitt, 2021; Sherr et al., 2022) | | |
| Increased caregiver engagement | fewer logistic barriers to attendance | “able to overcome barriers like childcare/transportation to the office.” (Barnett et al., 2021) |
|  | more opportunities to reinforce key messages | “I liked this structure and it was much understandable for me. I liked it when you sent something let’s say a picture and later on you sent also a voice note which had an additional explanation of the same thing, in fact, I was very happy because you were making us understand the lessons more, and also after that you were asking some questions to the participants and allow us to share our experiences, I really liked it, it was the best structure.” (du Toit et al., 2021) |
|  | more caregiver interaction | “I’m a very shy person and I wouldn’t normally talk in a group. I was able to say things to the (online) group.” (Cook et al., 2021) |
|  | more male engagement | “I feel the families are more open to—especially the moms—to communicating, but I think dads even. That’s a big success that we’re engaging more dads in the home visits.” (Ferrara et al., 2022) |
| Improving caregiver learning | learning in a natural environment | “Transfer of the parents’ skills learned in treatment is better than if they were in the clinic because they are learning/practicing learned skills in their natural environment.” (Barnett et al., 2021) |
|  | opportunities of independent problem-solving | “It was easier for the facilitator to encourage parents to wrestle with questions and work out for themselves where the child was on the circle, what the child needs from the parent in the moment, and most importantly to see that meeting that need is not only possible but rewarding for both parent and child.” (Cook et al., 2021) |
|  | flexibility in receiving individualized and extra support | “This way, I get to see a small picture of their daily life and ask more nuanced questions such as ‘He’s moving his hands but is he really not listening to the story?’ ‘What do you think you need the most help with in order to get him to interact with you?’” (McDevitt, 2021) |
| Promoting facilitator professional growth | re-thinking about the program, group facilitation, and participants | “During this time where we’ve been at home, we’ve been able to grow so much professionally.” Succinctly put, “so, I’ve been doing a lot of learning.” (Ferrara et al., 2022) |
| Adjusting to the ‘new normal’ | Promoting program sustainability during and post-COVID19 | “’this whole experience is going to change how we go forward.’ One home visitor stated, ‘We’re going to have to adjust to a new normal. We have some of the tools right now, but we need training to be able to, like I said before about innovation, you have to be ready to change and willing to change.’” (Ferrara et al., 2022) |
| **Perceived Challenges of Digital Delivery**  **Studies that contribute to this theme:** (Barnett et al., 2021; Cook et al., 2021; du Toit et al., 2021; Ferrara et al., 2022; McDevitt, 2021; Sherr et al., 2022; Shenderovich et al., 2022) | | |
| Technical and resource issues | lack of devices, reliable internet access, and technology readiness | “Hard to see the child consistently during session as they wander off screen; hard to hear what is happening in the room and child’s statements as clearly as in-offfce; disruptions to technology (clients getting disconnected, headphones running out of battery, etc.).” (Barnett et al., 2021) |
|  | Privacy and online safety | “We did not have security because you’re using your personal laptop, so you do not have security features and that was a challenge.” (Ferrara et al., 2022) |
| Difficulty in providing full services | Difficulty in identifying violence against children | “And again, when it comes to the abuse or domestic violence piece of things, not having those real eyes on. ” (Ferrara et al., 2022) |
| Difficulty in program evaluation | \ | “getting parents to fill out electronic ECBI before sessions has been very difficult and it takes up a lot of time to fill out the questions with them at the beginning of session.” (Barnett et al., 2021) |
| Barriers to engaging participants | difficulties in remote communication | “I didn’t realize it was hard to sort of explain, for me, things over the phone. ” (Ferrara et al., 2022) |
|  | lack of structured setting | “Home environment (setup) is difficult for parents to manage and many parents are resistant to adapting their setups for a more efficient therapy session.” (Barnett et al., 2021) |
|  | more distractions | “Keeping parents engaged when they are distracted by siblings, taking calls, other service providers (e.g., exterminator) arriving. ” (Barnett et al., 2021) |
|  | limited acceptance of remote programs | “‘If they can’t have face-to-face, they don’t want visits.’ One aspect discussed was the number of clients lost during COVID-19 shutdowns: ‘Some of us lost probably half of our clients, some of us lost more, some of us lost a third. I mean, we had a large chunk of people just not engaging.’ Explanations for why they saw this trailing off were noted: ‘As time went on, those [responses] start slowing down as well. And a lot of them, they would tell me, if I need anything, I will reach out to you.’” (Ferrara et al., 2022) |
| **Stakeholder Suggestions for Improvement**  **Studies that contribute to this theme:** (Cook et al., 2021; du Toit et al., 2021; Ferrara et al., 2022; Sherr et al., 2022) | | |
| New program structures | smaller group size | “A large proportion of caregivers suggested that smaller groups (less than 20 caregivers) would work better and would be more conducive for participation. Nearly every caregiver who was interviewed asked if the programme could continue for longer than the initial six weeks.” (du Toit et al., 2021) |
|  | add-on in-person elements | “Caregivers noted that meeting face-to-face would be beneficial as an add-on to the digital programme, suggesting an initial meeting at the beginning of the programme before receiving the digital programme to meet the facilitators and other members of the groups. ” (du Toit et al., 2021) |
| Boundaries with caregivers | \ | “‘Sometimes they will call at 10 o’clock at night and stuff like that. So, I’ve had to kind of create boundaries.’ Another home visitor said, ‘For whatever reason, my clients thought, oh, well, she’s from home now. She said I could text whenever, so it loosens the boundaries.’” (Ferrara et al., 2022) |
| Organizational and wider support | \ | “When I have gone to management, I feel like they’re backed up so much that I’m left hanging…and, it becomes, kind of feeling a little frustrated.” “Just more guidance and direction on what we’re supposed to do. I mean, that was just lacking” (Ferrara et al., 2022)  “I think one positive thing that I appreciated was allowing people to speak into your process to say here could you try this out. And then that feedback is included into the process that I appreciated that.” (Sherr et al., 2022)  “‘families that are still needing outside services like therapy or help with enrollment for [other services].’ Not only providing referrals to these services, but also connecting with those service providers was needed: ‘I have had a couple of cases where I’ve connected with another program. For instance, reaching out to the [Exceptional Family Members Program] after I’ve had a conversation with one of my clients who needed connection there and help[ed] to close that loop.’” (Ferrara et al., 2022) |

# Appendix 3 Cross-Referencing Tables

| **STUDY** | **DESIGN** | **OUTCOME** | **ADAPTATION** |  |
| --- | --- | --- | --- | --- |
| 1. **Violence against children** | | | | |
| Amaral 2022 | quantitative: RCT (control: treatment-as-usual) | No significant group differences | The 27 intervention topics were delivered as messages via SMS or WhatsApp weekly. |  |
| 1. **Child behaviors** | | | | |
| Canário 2021 | quantitative: descriptive (pre-post) qualitative: semi-structured interviews | improvement in 3 children | moved online; changes to exercises and activities (breakdown of changes in each session in supplementary materials), use of breakout rooms; summary of supplementary material - list each exercise and the adjustments made to the way the program is delivered to suit online format (e.g., breakout rooms, process for doing introductions, paper/pencil activity in small groups changed to big group, etc.) |  |
| Garcia | quantitative: descriptive (pre-post) | Reduced child externalizing and internalizing problems  Improved child compliance | both virtual and in-person delivery; Therapists coach caregivers on their parenting skills from behind a one-way mirror via a wireless headset (for in-person services) or via videoconferencing (for virtual services).; table 2 summarizes virtual implementation strategies - web conference training from outside agencies; recorded trainings developed by PCIT team; one-on-one consultation; skills practice; shadowing cases; reviewing cases; FAQ document; online community of practice; live observation and feedback; virtual training materials (I-PCIT guide); in-session co-therapist support |  |
| Gerow | quantitative: descriptive (concurrent multiple-baseline) | Reduced child externalizing and internalizing problems | move to telehealth coaching, also self-directed components; new equipment and procedures to deliver sessions; mailed supplies to family homes; used Vsee to run sessions; |  |
| Melo 2021 | quantitative: descriptive (pre-post) qualitative: case study | Improved child behaviour | home-based internet-PCIT (I-PCIT) using nothing more than a cell phone with video capabilities that was connected to a videotelephony software program and set-up n the child’s home by the parent |  |
| Amaral 2022 | quantitative: RCT (control: treatment-as-usual) | No significant group difference | The 27 intervention topics were delivered as messages via SMS or WhatsApp weekly |  |
| du Toit 2021 | quantitative: RCT (control: waitlist) qualitative: semi-structured interviews | No significant group difference | adapted program material to be delivered via text messages, voice notes, infographics, animation videos, video clips, online Book Dash repository; increased number of participants reached per group (5-8 in person to then 30-40 online); content in-person groups to online via aforementioned mediums; changed to text message support group discussions from previously one to one practice sessions; change to receive two digital picture books a week over WhatsApp from in-person received a picture book to take home each week; online received recap of messages via text but in-person was take home card with key messages; received data bundles to support participation |  |
| Agazzi 2022 | quantitative: non-randomized controlled trial (control: in-person version) |  | With the onset of COVID-19, in-person DOCS K-5 sessions were suspended in favor of a telehealth version administered Microsoft Teams. Each i-DOCS K-5 group consisted of 10–15 caregivers and involved the same content as the in-person program as previously described.  Participant materials were mailed to caregivers’ homes, and additional handouts were emailed in PDF format. Caregivers also were provided with technology support as needed, such as helping them install and navigate the functions of Microsoft Teams. |  |
| Agazzi 2021 | quantitative: non-randomized controlled trial (control: in-person version) | No significant group differences | Due to COVID-19, in-person HOT DOCS was temporarily suspended in March 2020, and only i-HOT DOCS was delivered through a HIPAA compliant online meeting platform (Microsoft Teams).   Most activities were unchanged, with participants watching videos and engaging in group discussion through Microsoft Teams. When completing worksheets, participants were given a set amount of time to independently respond to items on the handout, before reviewing responses as a group with the instructor(s), whereas in in-person sessions, participants were paired with a partner to complete worksheets. Participant manuals were mailed to homes, and any additional handouts or materials were converted to PDF format and shared via e-mail or text message. Participants were offered technology support sessions prior to sessions if they were struggling to log in to the class. Telephone support included walking the participant through how to download Microsoft Teams... |  |
| 1. **Child development** | | | | |
| Canário 2021 | quantitative: descriptive (pre-post) qualitative: semi-structured interviews | Reduced BMI in 1 child | moved online; changes to exercises and activities (breakdown of changes in each session in supplementary materials), use of breakout rooms; summary of supplementary material - list each exercise and the adjustments made to the way the program is delivered to suit online format (e.g., breakout rooms, process for doing introductions, paper/pencil activity in small groups changed to big group, etc.) |  |
| du Toit 2021 | quantitative: RCT (control: waitlist) qualitative: semi-structured interviews | Group difference in child social and emotional development is not significant. | adapted program material to be delivered via text messages, voice notes, infographics, animation videos, video clips, online Book Dash repository; increased number of participants reached per group (5-8 in person to then 30-40 online); content in-person groups to online via aforementioned mediums; changed to text message support group discussions from previously one to one practice sessions; change to receive two digital picture books a week over WhatsApp from in-person received a picture book to take home each week; online received recap of messages via text but in-person was take home card with key messages; received data bundles to support participation |  |
| 1. **Belief in harsh parenting** | | | | |
| du Toit 2021 | quantitative: RCT (control: waitlist) qualitative: semi-structured interviews | No significant group difference | adapted program material to be delivered via text messages, voice notes, infographics, animation videos, video clips, online Book Dash repository; increased number of participants reached per group (5-8 in person to then 30-40 online); content in-person groups to online via aforementioned mediums; changed to text message support group discussions from previously one to one practice sessions; change to receive two digital picture books a week over WhatsApp from in-person received a picture book to take home each week; online received recap of messages via text but in-person was take home card with key messages; received data bundles to support participation |  |
| 1. **Parental mental health** | | | | |
| du Toit 2021 | quantitative: RCT (control: waitlist) qualitative: semi-structured interviews | Improved in Zambia but not in Tanzania | adapted program material to be delivered via text messages, voice notes, infographics, animation videos, video clips, online Book Dash repository; increased number of participants reached per group (5-8 in person to then 30-40 online); content in-person groups to online via aforementioned mediums; changed to text message support group discussions from previously one to one practice sessions; change to receive two digital picture books a week over WhatsApp from in-person received a picture book to take home each week; online received recap of messages via text but in-person was take home card with key messages; received data bundles to support participation |  |
| Liu 2021 | quantitative: non-randomized controlled trial (control: treatment-as-usual) | Reduced parental anxiety, depression, and stress;  Increased parental sense of hope | The Joint Attention, Symbolic Play, Engagement, and Regulation (JASPER) online course delivered via WeChat [13]. The JASPER course focused on targeted social communication strategies in the format of parent-child coaching sessions that went on for 45-60 minutes per session, with two sessions each week for 12 weeks. Specific strategies for high-quality responses to children’s communication and behaviors were provided by one special training teacher with more than five years of special training work experience. Another teacher was responsible for demonstrating any scenario simulations. An online question-and-answer session. A question-and-answer session (30-40 minutes) was conducted each week for 12 weeks. An online parental psychological intervention course based on pandemic situations. The course was conducted by team researchers with second-level psychological counseling qualifications. The contents included home protection strategies, emotional management, parental stress coping strategies, and psychological counseling strategies to cope with the pandemic situation (e.g., mindfulness breathing training, muscle relaxation training, and the traditional Chinese Qigong exercise “Ba Duan Jin”) and lasted 45-60 minutes per session, with one session every two weeks and 6 sessions in total. For all online courses, live links were generated by the class assistant software Little Goose (Shenzhen Xiao’e Network Technology Co) and then sent to the WeChat group. |  |
| Garcia | quantitative: descriptive (pre-post) | Reduced parenting stress | both virtual and in-person delivery; Therapists coach caregivers on their parenting skills from behind a one-way mirror via a wireless headset (for in-person services) or via videoconferencing (for virtual services).; table 2 summarizes virtual implementation strategies - web conference training from outside agencies; recorded trainings developed by PCIT team; one-on-one consultation; skills practice; shadowing cases; reviewing cases; FAQ document; online community of practice; live observation and feedback; virtual training materials (I-PCIT guide); in-session co-therapist support |  |
| Traube 2022 | quantitative: descriptive (cross-sectional survey) | Reduced parental depression and anxiety | virtual home visitation” (VHV) service delivery |  |
| Agazzi 2022 | quantitative: non-randomized controlled trial (control: in-person version) | No significant group difference | With the onset of COVID-19, in-person DOCS K-5 sessions were suspended in favor of a telehealth version administered Microsoft Teams. Each i-DOCS K-5 group consisted of 10–15 caregivers and involved the same content as the in-person program as previously described.   Participant materials were mailed to caregivers’ homes, and additional handouts were emailed in PDF format. Caregivers also were provided with technology support as needed, such as helping them install and navigate the functions of Microsoft Teams. |  |
| Agazzi 2021 | quantitative: non-randomized controlled trial (control: in-person version) | No significant group difference | Due to COVID-19, in-person HOT DOCS was temporarily suspended in March 2020, and only i-HOT DOCS was delivered through a HIPAA compliant online meeting platform (Microsoft Teams).   Most activities were unchanged, with participants watching videos and engaging in group discussion through Microsoft Teams. When completing worksheets, participants were given a set amount of time to independently respond to items on the handout, before reviewing responses as a group with the instructor(s), whereas in in-person sessions, participants were paired with a partner to complete worksheets. Participant manuals were mailed to homes, and any additional handouts or materials were converted to PDF format and shared via e-mail or text message. Participants were offered technology support sessions prior to sessions if they were struggling to log in to the class. Telephone support included walking the participant through how to download Microsoft Teams... |  |
| Amaral 2022 | quantitative: RCT (control: treatment-as-usual) | Increased parental mental distress, especially among male caregivers.  No significant group difference in anxiety and depression. | The 27 intervention topics were delivered as messages via SMS or WhatsApp weekly |  |
| 1. **Parenting style** | | | | |
| du Toit 2021 | quantitative: RCT (control: waitlist) qualitative: semi-structured interviews | Increased responsive caregiving  Increased time in reading, looking at picture books and/telling their child stories | adapted program material to be delivered via text messages, voice notes, infographics, animation videos, video clips, online Book Dash repository; increased number of participants reached per group (5-8 in person to then 30-40 online); content in-person groups to online via aforementioned mediums; changed to text message support group discussions from previously one to one practice sessions; change to receive two digital picture books a week over WhatsApp from in-person received a picture book to take home each week; online received recap of messages via text but in-person was take home card with key messages; received data bundles to support participation |  |
| Canário 2021 | quantitative: descriptive (pre-post) qualitative: semi-structured interviews | Improved overall parenting style, feeding practices, and physical activity encourage time. | moved online; changes to exercises and activities (breakdown of changes in each session in supplementary materials), use of breakout rooms; summary of supplementary material - list each exercise and the adjustments made to the way the program is delivered to suit online format (e.g., breakout rooms, process for doing introductions, paper/pencil activity in small groups changed to big group, etc.) |  |
| Garcia | quantitative: descriptive (pre-post) | Increased positive parenting | both virtual and in-person delivery; Therapists coach caregivers on their parenting skills from behind a one-way mirror via a wireless headset (for in-person services) or via videoconferencing (for virtual services).; table 2 summarizes virtual implementation strategies - web conference training from outside agencies; recorded trainings developed by PCIT team; one-on-one consultation; skills practice; shadowing cases; reviewing cases; FAQ document; online community of practice; live observation and feedback; virtual training materials (I-PCIT guide); in-session co-therapist support |  |
| Gerow | quantitative: descriptive (concurrent multiple-baseline) | Increased positive parenting skills | move to telehealth coaching, also self-directed components; new equipment and procedures to deliver sessions; mailed supplies to family homes; used Vsee to run sessions; |  |
| Lewis 2022 | quantitative: descriptive (pre-post) | Improved parental reflective functioning  Increased parental self-efficacy | The group content was delivered in several bespoke formats to meet the needs of those attending. For example, some groups followed a six two-hour session plan (based upon Kim Golding’s group), whilst others the content was delivered in two six-hour sessions. Adaptations were made to account for meeting virtually. These included: more frequent breaks; offering additional technical support for those requiring it; using virtual breakout rooms for small group discussions; using a range of modalities to deliver group content such as YouTube video clips and Canva slides |  |
| Amaral 2022 | quantitative: RCT (control: treatment-as-usual) | No significant group differences | The 27 intervention topics were delivered as messages via SMS or WhatsApp weekly |  |
| Schein 2022 | quantitative: non-randomized controlled trial (control: hybrid delivery) | No significant group differences | Using video conferencing to provide observations of live parent-child interactions. |  |
| 1. **Parent-child interaction** | | | | |
| Amaral 2022 | quantitative: RCT (control: treatment-as-usual) | No significant group difference among female caregivers, but negative impact among male caregivers | The 27 intervention topics were delivered as messages via SMS or WhatsApp weekly |  |
| Melo 2021 | quantitative: descriptive (pre-post) qualitative: case study | Improved parent-child interaction | home-based internet-PCIT (I-PCIT) using nothing more than a cell phone with video capabilities that was connected to a videotelephony software program and set-up n the child’s home by the parent |  |
| 1. **Participant** **engagement** | | | | |
| Amaral 2022 | quantitative: RCT (control: treatment-as-usual) | Attendance rate: 72% | The 27 intervention topics were delivered as messages via SMS or WhatsApp weekly |  |
| Baggett | quant descriptive mapping of referrals before and during covid | Prior to the pandemic, 97% of study participants successfully progressed from consent to intervention, as compared to significant fewer (86%) during the pandemic. | how referrals took place; pre-pandemic was a mix of staff and self-referrals; pandemic transitioned to online self-referrals only |  |
| Canário 2021 | quantitative: descriptive (pre-post) qualitative: semi-structured interviews | Retention rate: 87.5%  Attendance rate: 92.86% | moved online; changes to exercises and activities (breakdown of changes in each session in supplementary materials), use of breakout rooms; summary of supplementary material - list each exercise and the adjustments made to the way the program is delivered to suit online format (e.g., breakout rooms, process for doing introductions, paper/pencil activity in small groups changed to big group, etc.) |  |
| du Toit 2021 | quantitative: RCT (control: waitlist) qualitative: semi-structured interviews | In Week 1 of the intervention, between 60-81% of caregivers in each group across all three countries had opened content within 24 hours of it being sent. By Week 6 of the intervention, this reduced to 30-76%. In Tanzania and Zambia, percentages did not drop below 50%, while in Uganda, percentages dropped to 30% in Week 6.  Figure 5 (page 46) illustrates the percentage of caregivers in each WhatsApp group who opened the message with the second digital book, sent on a Friday, within 24 hours. In the first week of the intervention, between 31-66% of caregivers across groups opened the Friday digital book within 24 hours, and by Week 6 of the intervention this increased to 44-86%. | adapted program material to be delivered via text messages, voice notes, infographics, animation videos, video clips, online Book Dash repository; increased number of participants reached per group (5-8 in person to then 30-40 online); content in-person groups to online via aforementioned mediums; changed to text message support group discussions from previously one to one practice sessions; change to receive two digital picture books a week over WhatsApp from in-person received a picture book to take home each week; online received recap of messages via text but in-person was take home card with key messages; received data bundles to support participation |  |
| Yi 2021 | quantitative: RCT (the same intervention but with modifications during the onboarding meeting and progress monitoring) | Higher retention and completion rates. For families in the ACT group, on average, they completed 64.29% of the online | online consultations |  |
| 1. **Participant** **satisfaction** | | | | |
| Agazzi 2021 | quantitative: non-randomized controlled trial (control: in-person version) | No significant difference between online and in-person versions in treatment satisfaction measure. | Due to COVID-19, in-person HOT DOCS was temporarily suspended in March 2020, and only i-HOT DOCS was delivered through a HIPAA compliant online meeting platform (Microsoft Teams).   Most activities were unchanged, with participants watching videos and engaging in group discussion through Microsoft Teams. When completing worksheets, participants were given a set amount of time to independently respond to items on the handout, before reviewing responses as a group with the instructor(s), whereas in in-person sessions, participants were paired with a partner to complete worksheets. Participant manuals were mailed to homes, and any additional handouts or materials were converted to PDF format and shared via e-mail or text message. Participants were offered technology support sessions prior to sessions if they were struggling to log in to the class. Telephone support included walking the participant through how to download Microsoft Teams... |  |
| Lo 2022 | quantitative: descriptive (post-test) qualitative: focus group discussions | High level of participant satisfaction (4/5) | changed from all delivery on site to hybrid or some onsite and some online sessions. Reduced the number of contact hours from 40 to 30. breakout rooms used and different strategies were adopted during the group sessions to maintain the attention of the participants, including doing stretching exercises to energize the group, using multisensory stimuli available on the internet (e.g., music and cartoons) |  |
| Agazzi 2022 | quantitative: non-randomized controlled trial (control: in-person version) | No significant difference between online and in-person versions in treatment satisfaction measure. | With the onset of COVID-19, in-person DOCS K-5 sessions were suspended in favor of a telehealth version administered Microsoft Teams. Each i-DOCS K-5 group consisted of 10–15 caregivers and involved the same content as the in-person program as previously described.   Participant materials were mailed to caregivers’ homes, and additional handouts were emailed in PDF format. Caregivers also were provided with technology support as needed, such as helping them install and navigate the functions of Microsoft Teams. |  |
| McIntyre 2022 | quantitative: descriptive (post-test) | 75% consider the online programme acceptable  91% think it is good to have online programme  66% reported easy to learn the information online | Each session was conducted using Zoom Video Communications, a cloud-based peer to peer software platform used for videotelephony and online chat services. All sessions were facilitated in Spanish by two group leaders. Two bilingual research assistants were also present in order to provide technology support and assess intervention fidelity. A bilingual BPT supervisor also attended sessions or watched recorded sessions every week. Each session was structured around videotape vignettes (using Webster-Stratton’s original content with Spanish subtitles and translated material; see Webster-Stratton, 2001) and used discussion, modeling, and feedback techniques to foster mastery of the presented materials |  |
| 1. **Program fidelity** | | | | |
| Caron | quantitative: descriptive (fidelity scores) | Providers demonstrated improved fidelity over the course of training.  When in-person and telehealth-delivered sessions were compared, providers’ fidelity in telehealth-delivered ABC sessions was not significantly different from their fidelity in in- person sessions.  Providers demonstrated improved fidelity over time in telehealth-delivered sessions. | moved to telehealth sessions during pandemic, and then did a mix of in-person and telehealth afterwards |  |
| Gerow | quantitative: descriptive (concurrent multiple-baseline) | Parent implementation fidelity and therapist coaching fidelity were both high. | move to telehealth coaching, also self-directed components; new equipment and procedures to deliver sessions; mailed supplies to family homes; used Vsee to run sessions; |  |
| Roben 2022 | quantitative: descriptive (process measures) | High fidelity rate of 83.33% | Using video conferencing to provide observations of live parent-child interactions. |  |
| du Toit 2021 | quantitative: RCT (control: waitlist) qualitative: semi-structured interviews | High fidelity | adapted program material to be delivered via text messages, voice notes, infographics, animation videos, video clips, online Book Dash repository; increased number of participants reached per group (5-8 in person to then 30-40 online); content in-person groups to online via aforementioned mediums; changed to text message support group discussions from previously one to one practice sessions; change to receive two digital picture books a week over WhatsApp from in-person received a picture book to take home each week; online received recap of messages via text but in-person was take home card with key messages; received data bundles to support participation |  |
| McIntyre 2022 | quantitative: descriptive (post-test) | Overall, the treatment adherence was high. In the BPT-M condition, all of the 120 BPT intervention elements were implemented (100% adherence). In the BPT-E condition, 117 BPT intervention elements were implemented (97.5% adherence). The difference in treatment adherence scores was not significantly different between conditions (t [18] = −0.24, p > .05), 95% CI [-2.92, 2.32], d =.11. Average contact time, or dosage, for telehealth groups in the BPT-M condition was 88.10minutes (SD=4.07) and 87.80minutes (SD=4.42) for the BPT-E group. The difference in dosage between conditions was not significantly different (t [18] = −0.16, p > .05), 95% CI [-4.29, 3.69], d =.07 | Each session was conducted using Zoom Video Communications, a cloud-based peer to peer software platform used for videotelephony and online chat services. All sessions were facilitated in Spanish by two group leaders. Two bilingual research assistants were also present in order to provide technology support and assess intervention fidelity. A bilingual BPT supervisor also attended sessions or watched recorded sessions every week. Each session was structured around videotape vignettes (using Webster-Stratton’s original content with Spanish subtitles and translated material; see Webster-Stratton, 2001) and used discussion, modeling, and feedback techniques to foster mastery of the presented materials |  |

# Appendix 4 List of Databases and Grey Literature Repositories

English databases:

1. MEDLINE

2. Embase

3. PsycINFO

4. Cochrane library

5. CINAHL

6. Applied Social Sciences Index and Abstracts

5. Education Resources Information Center

6. International Bibliography of the Social Sciences

7. Social Science Premium Collection

Chinese databases:

1. China National Knowledge Infrastructure (CNKI)

2. China Science and Technology Journal Database (CSTJ)

3. Wanfang Database

Grey literature repositories:

1. ProQuest Dissertations & Theses Global

2. Clinical Trials.gov

3. World Health Organization clinical trials

4. UNICEF Office of Research – Innocenti

5. WHO Global Health Library--The Western Pacific Region Index Medicus (WPRIM)

6. International Development Research Centre (IDRC)

# Appendix 5 Sample Search Strategies

Embase, PsycINFO, Medline:

1. ((parent$ or famil$ or caregiver$ or caretaker$) adj2 (program$ or intervention$ or training or education or group$ or coach$)).ti,ab,kw.

2. (behavio#r adj3 (train$ or intervention$ or therap$ or program$)).ti,ab,kw.

3. (cbt or cognitive behavio#ral therapy).ti,ab,kw.

4. (cognitive adj3 (therap$ or intervention$ or train$ or program$)).ti,ab,kw.

5. (triple p or positive parenting program$).ti,ab,kw.

6. incredible years.ti,ab,kw.

7. (pcit or ipcit or i-pcit or (parent-child interaction adj therap$) or (Parent-Child Interaction adj Therap$)).ti,ab,kw.

8. (pmt or (parent adj management adj training)).ti,ab,kw.

9. (family adj check-up).ti,ab,kw.

10. exp parenting/

11. (adapt$ or modif$ or optimi$ or adjust$ or tailor$ or alterat$ or develop$ or deliver$ or implement$ or remote$ or digital$ or internet$ or online$ or virtual$).ti,ab,kw.

12. (COVID* or corona* or SARS-COV* or pandemic or lockdown or epidemic).af

13. 1 or 2 or 3 or 4 or 5 or 6 or 7 or 8 or 9 or 10

14. 11 and 12 and 13

15. limit 14 to yr="2020 -Current"
